# IntelliCage Automated Behavioral Phenotyping Reveals Behavior Deficits in the 3xTg-AD Mouse Model of Alzheimer’s Disease Associated With Brain Weight

**DOI:** 10.3389/fnagi.2021.720214

**Published:** 2021-08-13

**Authors:** Wendy Winslow, Ian McDonough, Savannah Tallino, Annika Decker, Austin S. Vural, Ramon Velazquez

**Affiliations:** ^1^Arizona State University-Banner Neurodegenerative Disease Research Center at the Biodesign Institute, Arizona State University, Tempe, AZ, United States; ^2^School of Life Sciences, Arizona State University, Tempe, AZ, United States; ^3^Arizona Alzheimer’s Consortium, Phoenix, AZ, United States

**Keywords:** IntelliCage, 3xTg-AD, brain weight, cognition, amyloidosis

## Abstract

Transgenic rodent models of Alzheimer’s disease (AD) were designed to study mechanisms of pathogenesis and connect these mechanisms with cognitive decline. Measurements of cognition in rodents can be confounded, however, by human handling and interaction; the IntelliCage was created to circumvent these issues while measuring various facets of cognition in a social environment with water consumption as the primary motivator for task completion. Here, for the first time, we examined the behavioral performance of 3xTg-AD mice in the IntelliCage. Seven- to 9-month-old female 3xTg-AD and non-transgenic (NonTg) mice were tested for 29 days in the IntelliCage to measure prefrontal cortical and hippocampal function. We found that a higher percentage of NonTg mice (86.96%) were able to successfully complete the training (adaptation) phases compared to their 3xTg-AD (57.14%) counterparts. Furthermore, the 3xTg-AD mice showed impairments in attention and working memory. Interestingly, we found that differences in body and brain weight between NonTg and 3xTg-AD mice were associated with whether mice were able to complete the IntelliCage tasks. 3xTg-AD mice that completed IntelliCage tasks had lower cortical insoluble amyloid-β_40_ fractions than their 3xTg-AD counterparts who failed to complete the tasks. Collectively, these results demonstrate deficits in cognition in the 3xTg-AD mouse and inform scientists of important factors to consider when testing this transgenic model in the IntelliCage.

## Introduction

Alzheimer’s disease (AD) is the most common cause of dementia and is a top health concern globally; in the US alone, it is projected to rise from 5 million cases currently to ~15 million by 2050, with national costs rising as high as $1.1 trillion (Alzheimer’s association report, 2021). Clinically, AD presents with cognitive impairment including deficits in new memory development, loss of long-term memories that worsen as the disease progresses, and a severe loss of general intellectual ability coinciding with dementia (Honjo et al., 2012; Bateman, 2015). Key neuropathological features of AD include extracellular plaques of the amyloid-β (Aβ) peptide, neurofibrillary tangles (NFTs)—intraneuronal tangles of hyperphosphorylated tau–and synaptic and neural loss (Honjo et al., 2012; Bakota and Brandt, 2016; Lane et al., 2018). Therapeutic options remain extremely limited for advanced AD, highlighting the need for preclinical research into molecular events preceding Aβ and NFT pathology prior to dementia onset.

Aβ plaques observed in AD are derived from the Aβ peptide, which ranges from 36 to 43 amino acids in length. Aβ_40_ and Aβ_42_ are the most abundant Aβ species observed in AD (O’Brien and Wong, 2011; Sadigh-Eteghad et al., 2015). The shift from soluble to insoluble forms of Aβ_40_ and Aβ_42_ has been identified as what may distinguish dementia pathology from normal aging (Wang et al., 1999). It has been shown that Aβ_40_ plays a mechanistic role in the onset and/or progression of AD (Wang et al., 1999), and while insoluble Aβ_40_ is more prevalent than Aβ_42_, insoluble Aβ_42_ is more prone to aggregation and toxicity (O’Brien and Wong, 2011). The AD brain is also characterized by tau phosphorylation and NFTs (Braak et al., 2006). Numerous reports have highlighted that phosphorylation at specific tau sites, in particular, Ser202/Thr205 (AT8) and Thr214/Ser212 (AT100), is highly associated with increased intraneuronal filaments and neurodegeneration (Allen et al., 2002; Augustinack et al., 2002). Braak Staging, a well-established method that scores the accumulation of phospho-tau, is based on AT8 antibody staining (Braak and Braak, 1991; Braak et al., 2006); researchers have also identified the sequential appearance of specific tau phospho-dependent epitopes, revealing that AT100 phosphorylation appears after AT8 in human AD post-mortem brain tissue (Luna-Muñoz et al., 2007).

AD rodent models remain a key tool for unraveling pathogenic mechanisms and for the development of preclinical therapeutic options; 205 mouse models possessing many of the aspects of human AD are in use worldwide as of 2021 (Alz Forum, 2021). These models are developed by incorporating key human transgenes associated with familial AD into the mouse genome-mice do not spontaneously develop AD (Borchelt et al., 1997; Jankowsky et al., 2004; Reiserer et al., 2007; Hall and Roberson, 2012; LaFerla and Green, 2012). One of the most widely-used AD models that recapitulates hallmark Aβ and NFT pathologies is the 3xTg-AD mouse model, developed in 2003 (Oddo et al., 2003). This model incorporates the APP human Swedish mutation, a presenilin knock-in mutation, and a human P301L mutation. Aβ deposits are present by 6 months of age in the frontal cortex and become more extensive by 12 months of age in this model (Oddo et al., 2003; Sterniczuk et al., 2010). AT8 tau phosphorylation is widespread in the hippocampus by 6 months of age, while AT100 tau phosphorylation is more widespread in the hippocampus by 12 months (Oh et al., 2010; Parachikova et al., 2010). Spatial cognition and memory deficits have been documented as early as 6 months and become more pronounced by 12 months of age in the Morris water maze (MWM) spatial cognition task (Parachikova et al., 2010; Roda et al., 2020). Notably, reports have shown that brain weight in 15-month-old 3xTg-AD mice is reduced compared to NonTgs (Di Benedetto et al., 2019). Brain size is highly relevant and underexplored in the 3xTg-AD mice, as studies highlight that brain size may be associated with poorer cognitive function (Perepelkina et al., 2020).

Behavioral tasks have been developed to evaluate cognition in rodents, including in AD mouse models. However, these behavioral tasks rely on the daily handling of rodents while being transported to the testing apparatuses and during testing itself. Such testing protocols also contain other caveats, including experimental variability due to environmental factors, animal isolation, unnatural incentives to participate (e.g., water escape), and human errors in scoring. In 2000, Dr. Hans-Peter Lipp developed the IntelliCage, which helps overcome these factors by allowing rodents to be tested in their natural social environment with minimal human intervention (Dell’Omo et al., 2000; Lipp, 2005; Lipp et al., 2005). The IntelliCage is a fully-automated system allowing animals to engage in a wide variety of experimental tasks, using access to water as their incentive to participate, and has since been used in over 150 studies (Lipp et al., 2005; Lipp, 2005; Masuda et al., 2018; Kiryk et al., 2020; Mifflin et al., 2021). Animals can be assessed for exploratory behavior, water consumption patterns, spatial learning, behavioral flexibility, attention, impulsivity, and working and contextual memory within a single cage. While APP/KI and APP/PS1 mouse models of amyloidosis have been tested in the IntelliCage, to date, the 3xTg-AD model has not been evaluated in this automated behavioral phenotyping apparatus (Ryan et al., 2013; Lee et al., 2015; Masuda et al., 2016; Mifflin et al., 2021).

The goal of the present work was to test the 3xTg-AD mouse for the first time in the IntelliCage and determine if cognitive deficits can be detected. Additionally, we aimed to determine whether AD-like pathologies in 3xTg-AD mice-brain size, body weight, Aβ, and pathological tau-are associated with behavioral results from various phases of IntelliCage testing. Given previous reports relating brain size, Aβ, and pathological tau with cognition (Sterniczuk et al., 2010; Perepelkina et al., 2020), we hypothesized that increased presentation of these pathologies would be associated with deficits in the IntelliCage tasks.

## Materials and Methods

### Animals

3xTg-AD mice were generated on a C57BL6/129Svj hybrid background as previously described (Oddo et al., 2003; Velazquez et al., 2019c). Since 3xTg-AD mice are homozygous for mutations in the APP, PS1, and MAPT genes, colonies are maintained by breeding homozygous 3xTg-AD mice to each other. Notably, 3xTg-AD males show large neuropathological variability, even between littermates, while females do not show such variability. Therefore, as in most recent studies using the 3xTg-AD mouse, we only included female mice. All protocols were approved by the Institutional Animal Care and Use Committee of Arizona State University and conform to the National Institutes of Health Guide for the Care and Use of Laboratory Animals. Mice were group housed (4 to 5 mice per cage) prior to being introduced into the IntelliCage. At 7–9 months of age, prior to IntelliCage testing, a radiofrequency identification transponder chip (RFID; Standard Microchip T-VA, DataMars, Switzerland and Troven, USA) was subcutaneously implanted into the dorso-cervical region under isoflurane inhalation anesthesia as previously described (Mifflin et al., 2021). The RFID chip allows for the identification of a mouse when it enters a corner of the IntelliCage system. Mice were allowed 1 week to recover and were then introduced into the IntelliCage.

Female 3xTg-AD (*n* = 21) and C57BL6/129Svj (*n* = 23; herein referred to as NonTg) mice were placed in the IntelliCage for assessment across a variety of tasks tapping hippocampal and prefrontal cortical function (Ajonijebu et al., 2018; Voikar et al., 2018; Kiryk et al., 2020). IntelliCage testing took a total of 29 days from adaptation to the place avoidance retention phase. Each IntelliCage holds up to 16 mice, and our lab currently has three cages allowing us to test all of the mice in one cohort. We introduced the following number of mice to each cage: cage one *n* = 8 3xTg-AD and *n* = 8 NonTg, cage two *n* = 7 3xTg-AD and *n* = 8 NonTg, and cage three *n* = 6 3xTg-AD and *n* = 7 NonTg mice. Mice were subsequently euthanized at 8–10 months of age, and tissue was extracted and prepared for ELISAs and western blot analysis.

### Automated IntelliCage Testing

The IntelliCage was used to evaluate water drinking, exploratory behavior, spatial learning, reference memory, behavioral flexibility, attention, and contextual memory (Masuda et al., 2016, 2018; Kiryk et al., 2020; Mifflin et al., 2021). The testing apparatus (39 cm × 58 cm × 21 cm) contains four corner chambers accessible through an antenna-equipped open tunnel. A computer management system is used to regulate water access *via* two individual doors in each corner. A scanner, located at each corner entrance, registers each animal’s entrance by scanning the individual RFID; an animal’s entire body must enter the corner to register the RFID and for a visit to be counted. Nosepokes and licks are detected by sensors on the nose port and waterspout, respectively. Mice were fed *ad libitum* during the entire duration in the IntelliCage with standard mouse chow, and lights were on in the behavior room from 06:00–20:00. A video camera was placed outside the IntelliCage and recorded the entire testing session (24 h/7 days a week). The sequence of experimental behavioral tasks in the IntelliCage was as follows: (1) Adaptation, consisting of free adaptation, door adaptation, and nosepoke adaptation phases; (2) Place preference and reversal; (3) Serial reaction time; and (4) Place avoidance. Any animal that failed to consume water in a 24-h period was removed from the IntelliCage and placed in a standard cage for 7 h to avoid severe dehydration. If these animals were re-introduced into the IntelliCage and again failed to consume water, they were removed from the experiment.

Data were extracted using the TSE IntelliCagePlus Analyzer software, from which we exported the data into multiple tab-delimited text files. Then, using a Python script, the text files were converted into a single SQLite3 database file. Using this file as input, several Python scripts were then used to query the database using SQL to extract the number of visits, nosepokes and licks, in addition the visits with at least one nosepoke to each corner, visits with at least one lick to each corner, delay to first visit, delay to the first nosepoke, and delay to the first drink for each animal. The same script also sliced the data into 24-h periods and separated the data into Excel spreadsheets with the data for each day. For each task, the dependent variable calculated is described below.

(1) Adaptation Phases

- During the first 3 days of the adaptation phase (free adaptation) all the doors were open allowing free access to the water bottles, thereby acclimating mice to the new environment. During the next 3 days (door adaptation), the doors to the water bottles were closed but opened for any visit into the corner. The following were calculated for the free adaptation phase:
- Total visits (to measure exploratory and water-seeking behavior)
- Total licks (to measure water consumption)

For the last 3 days (nosepoke adaptation), doors were closed and could be opened with a nosepoke in a corner, thereby training animals to nosepoke to retrieve water. In addition to total visits and total licks, the following were calculated:

- Number of visits with ≥1 nosepoke (indicating adaptation/learning)
- Number of visits with ≥1 lick over total visits (indicates the number of visits due to water-seeking motivation instead of exploratory behavior)

(2) Place Preference and Reversal

- During the place preference phase, water was accessible in only one of the four corners for each of the mice. The correct corner for each mouse was chosen based on their previous visit habits, selecting among the least-visited corners to eliminate preferential corner bias. For the first 6 days, water was available only in the selected reward corner (place preference). For the last 6 days, water was available only in the opposite corner (reversal). To prevent overcrowding of the corners and learning by imitation, the selected reward corners were balanced by the number of mice and genotype, limiting the number to four per corner and 50–50 proportion of 3xTg-AD and NonTg genotypes. In addition to measuring total visits and licks per day, calculations for this task were as follows:
- % correct = (number of visits to correct corner)/(total corner visits)
- Correct visit with a lick = (number of visits to correct corner with nosepoke and ≥1 lick)/(total visits to correct corner)

(3) Serial Reaction Time (SRT) Attention task

- During the 3 days of the SRT task, when an animal entered an assigned corner (the correct corner from the prior place preference reversal task), the first nosepoke for a visit initiated a trial. A 2 s pre-cue delay was imposed prior to the illumination of a green LED, requiring mice to learn to wait for the LED. The green LED was turned on for 7 s, requiring the animal to nosepoke within this time frame to count as a correct response, resulting in the door opening and allowing access to water. A nosepoke during the 2 s pre-cue delay was counted as a premature response and is a measure of impulsivity. If the animal failed to nosepoke during the 7 s time frame, the LED turned off and the trial would reset, requiring the animal to restart the trial. In addition to measuring total visits and licks per day, calculations for this task were as follows:
- % initiated trials = (number of correct visits with a first nosepoke)/(total visits)
- % correct = (number of correct visits with nosepoke and lick)/(total visits)
- % incorrect = (number of correct visits without a nosepoke and lick)/(total visits)
- % premature = (number of correct visits with a response during the 2 s pre-cue delay)/(number of initiated trials)
- Reaction time = time (s) to extinguish LED during correct visits with a nosepoke

(4) Place Avoidance

- The place avoidance tasks included both training and probe trials. For day 1, 24-h training trial (learned avoidance), nosepoking in the reward corner administered an aversive air puff (~0.8 bar, 1 s air-puff). The doors in all corners remained closed and water was not available during the learned avoidance phase. In addition to measuring total visits per day, we also analyzed the number of corner visits with nosepokes at the air puff corner to assess working memory errors. After the 24-h training trial, the mice were moved to their standard home cages for a 24-h delay with water *ad libitum*. After the delay, the mice were reintroduced to the IntelliCage for 3 days with water available at all four corners and the air puff stimulus removed to assess retention and extinction. The data for retention and extinction was quantified as the % correct visits with nosepokes over total visits for each day.

### Brain Tissue Processing, ELISA, Western Blots, and Immunohistochemistry

At the completion of IntelliCage testing, mice body weights were recorded, and their brain was extracted and weighed. One hemisphere had the hippocampus and cortex dissected out and flash-frozen while the contralateral hemisphere was fixed in a glass vial of 4% paraformaldehyde for 48 h and then transferred into 0.02% sodium azide in phosphate-buffered saline until sectioning. 50-μm-thick free-floating sections were subsequently obtained using a vibratome and used for histology. Flash-frozen tissue was homogenized in a T-PER tissue protein extraction reagent supplemented with protease (Roche Applied Science, IN, USA) and phosphatase inhibitors (Millipore, MA, USA). The homogenized tissues were centrifuged at 4°C for 30 min, and the supernatant (soluble fraction) was stored at −80°C. We then homogenized the pellet in 70% formic acid followed by centrifuging at 4°C for 30 min. Hippocampal and cortical soluble and insoluble fractions of Aβ_40_ and Aβ_42_ were detected using the commercially available ELISA kits (Invitrogen-ThermoFisher Scientific) as previously described (Velazquez et al., 2019a,b). Western blots were performed under reducing conditions as we previously detailed (Velazquez et al., 2016, 2019a). Quantitative analyses of the western blots were obtained by normalizing the intensity of the protein of interest with its own loading control β-actin, within each blot. Licor Image Studio software was used to quantify the intensity of the bands of interest. The experimenter was blinded to the group allocations.

Immunohistochemistry for AT8 was performed as we previously described (Dave et al., 2021). Images from three sections per mouse including dorsal, medial, and ventral hippocampus were taken with a Zeiss Axio Imager A1 using a 40× objective. Images were photomerged to rebuild the image, and AT8+ cell number was obtained using ImageJ. The experimenter was blinded to the group allocation.

### Antibodies

All the antibodies used in this study have been validated by the manufacturer for use in mouse tissue. The following antibodies were purchased from Thermo Fisher; AT8 (1:500 dilution, catalog #MN1020); AT100 (1:500 dilution, catalog #MN1060); β-actin (1:10,000 dilution, catalog #PA1-16889). The following antibody was purchased from MilliporeSigma, 6E10 (FL-APP 1:1,000 dilution, catalog #MAB1560).

### Statistical Analyses

ANOVA was used to examine the various IntelliCage data with repeated measures when applicable using StatView 5.0.1 (SAS Institute) and GraphPad Prism 8.1.2. Data that included repeated dependent measures were first assessed for sphericity using Mauchly’s tests, and no violations were found necessitating corrections. Bonferroni’s corrected *post hoc* tests were performed when a significant interaction was observed. Student’s unpaired *t*-tests were employed for comparison of 3xTg-AD mice when appropriate. Examination of descriptive statistics revealed no other violations of any assumptions that required the use of statistical tests other than the ones used. Significance was set at *p* < 0.05.

## Results

### A Higher Percentage of 3xTg-AD Mice Failed to Complete the Adaptation Phases of the IntelliCage

**Figure 1:**
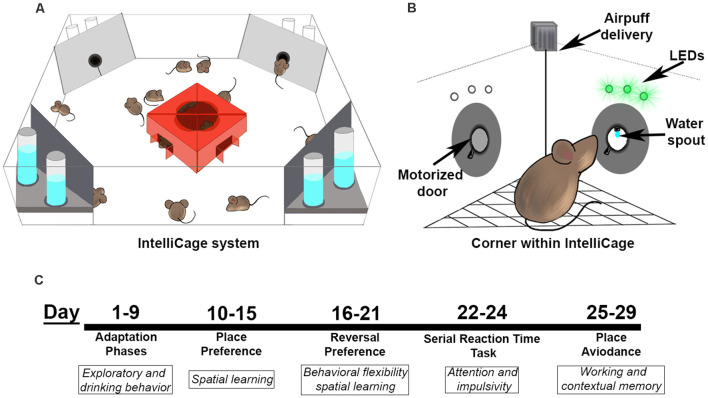
The IntelliCage and testing paradigm. **(A)** The IntelliCage accommodates up to 16 mice at a time and contains four corners that are programmed to allow water access based on nose port responses. **(B)** Each corner in the IntelliCage is equipped with two nose ports with doors, LEDs above each nose port, an air delivery nozzle, and access to water with a lickometer to detect drinking behavior. **(C)** Behavioral battery timeline for the IntelliCage study. Mice started testing at a range of 7–9 months of age.

**Figure 2:**
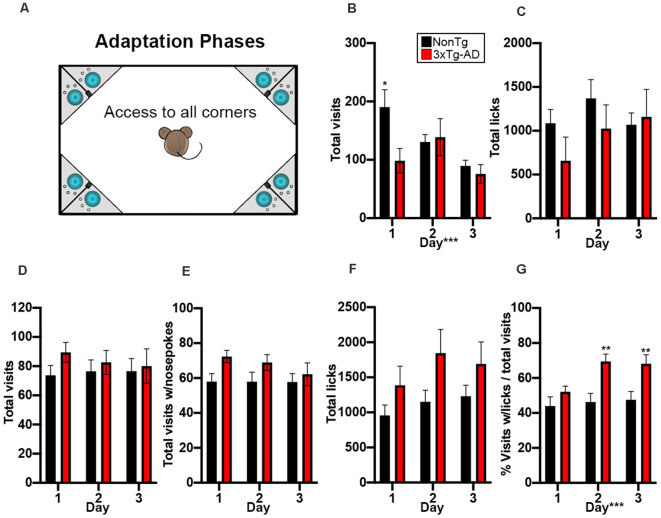
NonTg mice explore more and drink less than 3xTg-AD mice in the adaptation phases. **(A)** During the 3-day adaptation phase, animals freely entered any of the four corners and could access water: cage not drawn to scale. **(B)** We found that NonTg mice made more corner visits on day 1 compared to 3xTg-AD mice (*p* < 0.05). We also found that the number of visits decreased from day 1–3 (*p* < 0.001). **(C)** No differences detected in total licks. In the 3-day nosepoke adaptation phase, animals must learn to nosepoke to access water. **(D–F)** No significant differences were detected in total visits, total visits with a nosepoke, and total licks. **(G)** We found that 3xTg-AD mice made more total visits to lick than NonTg mice on days 2 and 3 of nosepoke adaptation, illustrating increased motivation to enter a corner and nosepoke to drink. Data are presented as means ± SEM. *p < 0.05, **p < 0.01, ***p < 0.001.

A total of 21 3xTg-AD mice and 23 NonTg mice were placed into the IntelliCage and started on the adaptation phases (Figures 1A–C). We found that a higher percentage of NonTg mice (86.96%) were able to make it past the adaptation phases compared to the 3xTg-AD counterparts (57.14%; Table 1); failure to pass the free adaptation phase was due to animals failing to drink. We next analyzed data for animals able to pass the free adaptation phase. During the 3 days of free adaptation, when all corners were accessible for water consumption (Figure 2A), we found a significant main effect of the day (*F*_(2,60)_ = 10.013, *p* < 0.001; Figure 2B), where total corner visits decreased across days. We also found a significant genotype by day interaction (*F*_(1,60)_ = 6.283, *p* < 0.01; Figure 2B); *post hoc* analysis revealed that NonTg mice made more corner visits than the 3xTg-AD mice on day 1 (*p* < 0.05). No differences in total licks were found during the free adaptation phase (Figure 2C), indicating that water consumption did not vary. Next, we analyzed data for the nosepoke adaptation phase, where animals had to nosepoke to receive water. We found no significant differences in total visits, total visits with a nosepoke, or total licks during the nosepoke adaptation phase (Figures 2D–F). To determine whether corner visits were due to exploration or water consumption, we analyzed the number of visits with ≥1 lick over total visits. We found a significant main effect of genotype (*F*_(1,60)_ = 6.869, *p* < 0.05; Figure 2G), where the 3xTg-AD entered corners to drink more frequently than the NonTg mice, despite both groups consuming the same volume of water (i.e., total licks). We also found a significant genotype by day interaction (*F*_(2,60)_ = 4.911, *p* < 0.05; Figure 2G). *Post hoc* analysis revealed that the 3xTg-AD entered corners to drink significantly more often than the NonTg mice on day 2 (*p* < 0.01) and 3 (*p* < 0.01). Collectively, these results indicate that a higher percentage of 3xTg-AD mice were not capable of learning to drink during the adaptation phases of the IntelliCage; those 3xTg-AD mice that did make it to the nosepoke adaptation phase entered corners frequently with the purpose of consuming water, while their NonTg counterparts’ motivation to enter corners varied between both exploration and water consumption.

**Table 1:** A higher percentage of NonTg mice were able to learn to enter a corner and drink from the waterspout compared to 3xTg-AD mice, resulting in a higher exclusion of 3xTg-AD mice from the subsequent IntelliCage tasks.

Genotype	Total start *n*	Total passed adaptation phase	% *n* passed adaptation
NonTg	23	20	86.96%
3xTg-AD	21	12	57.14%

*Nine 3xTg-AD and one NonTg mice failed to drink during Day 1–3 of adaptation. Two NonTg mice failed to drink during the door-adaption days (Day 4–7)*.

### 3xTg-AD Mice Performed Similarly to NonTg Mice in Learned Place Preference but Later Performed Better Than NonTg Mice in the Reversal Phase of the IntelliCage

**Figure 3:**
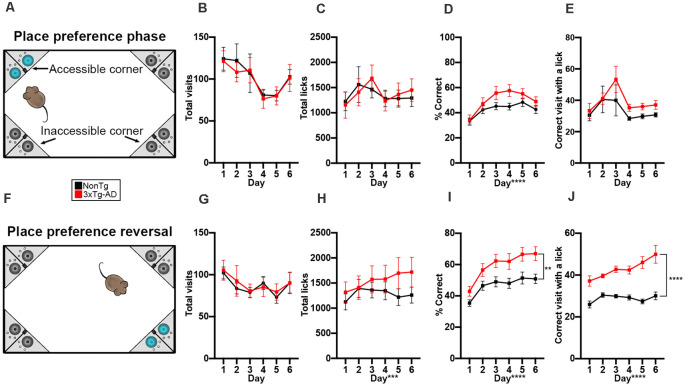
3xTg-AD mice performed similarly to NonTg mice in learned place preference but later performed better than NonTg mice in the reversal phase of the IntelliCage. **(A)** During the 6 days of the learned place preference phase, animals were assigned to one corner where they could access water. All other corners were counted as incorrect and allowed no access to water; cage not drawn to scale. **(B,C)** We found no significant differences in total visits and licks during the place preference phase. **(D)** We found a significant effect of day for % correct, illustrating learning across the 6 days, but no genotype differences were detected. **(E)** No differences were detected in the correct visits with ≥1 lick. **(F)** During the 6 days of the place preference reversal phase, animals could access water by entering and nosepoking the opposite corner from the corner assigned during the learned place preference phase; cage not drawn to scale. **(G)** No differences in total visits across the 6 days were detected. **(H)** We found that total licks increased across the 6 days (*p* < 0.001). **(I)** We found that 3xTg-AD had a higher % correct than NonTg (*p* < 0.01). Additionally, % correct increased across the 6 days (*p* < 0.0001), illustrating learning. **(J)** We found that 3xTg-AD mice made more visits to the correct corner with ≥1 lick than NonTg mice, illustrating that 3xTg-AD mice were motivated to enter the correct corner to drink. Additionally, correct corner visits with ≥1 lick increased across the 6 days (*p* < 0.0001). Data are presented as means ± SEM. **p < 0.01, ***p < 0.001, ****p < 0.0001.

During the learned place preference phase, animals were assigned to and only granted access to water from one corner (Figure 3A). Mice can use external environment cues to locate their correct corner, thereby assessing spatial learning (Ryan et al., 2013; Lee et al., 2015; Kiryk et al., 2020). We found no significant differences in total visits (Figure 3B) and total licks (Figure 3C), illustrating that both genotypes visited corners and drank equal amounts of water during this phase. We found a significant main effect of day for % correct in the place preference phase *F*_(1,140)_ = 19.063, *p* < 0.0001; Figure 3D), illustrating that performance improves across the 6 days for both genotypes. Lastly, to determine if animals visited the correct corner to drink or explore, we examined the visits to the correct corner with ≥1 lick. We found no significant differences, suggesting that in this phase, both genotypes’ motivation to enter the correct corner was at least in part due to water-seeking (Figure 3E).

Next, animals were assessed in the place preference reversal phase (Figure 3F), where the correct corner is opposite to that of the first phase of place preference. We found no significant differences in total visits between the NonTg and 3xTg-AD mice for this phase (Figure 3G). We did find a significant main effect of day for total licks (*F*_(1,140)_ = 4.450, *p* < 0.001, Figure 3H), illustrating increasing licking across the 6 days. For % correct, we found a significant main effect of day (*F*_(1,140)_ = 33.100, *p* < 0.0001; Figure 3I), illustrating learning across the 6 days. Surprisingly, we also found that the 3xTg-AD mice had a higher % correct in reversal preference phase than the NonTg mice (*F*_(1,140)_ = 7.767, *p* < 0.01; Figure 3I). When we examined the number of visits to the correct corner with ≥1 lick, we found a significant main effect of genotype (*F*_(1,140)_ = 64.107, *p* < 0.0001; Figure 3J), where the 3xTg-AD mice made a higher number of correct corner visits with ≥1 lick than the NonTg mice. We also found a significant main effect of day (*F*_(1,140)_ = 6.414, *p* < 0.0001), illustrating increased correct visits with ≥1 lick across the 6 days. Together, these results suggest that there are no differences in the early phase of preference learning between NonTg and 3xTg-AD mice; however, once animals learn the task, the 3xTg-AD mice made more correct visits to access water than the NonTg mice during place preference reversal. The number of visits to the correct corner with ≥1 lick was higher in 3xTg-AD compared to NonTg, suggesting that later in the place preference tasks, water seeking was the stronger motivator for corner visits among the 3xTg-AD mice while exploration played a dominant motivational role in NonTg mice.

### 3xTg-AD Mice Showed Impairments in Attention in the Serial Reaction Time (SRT) Tasks

**Figure 4:**
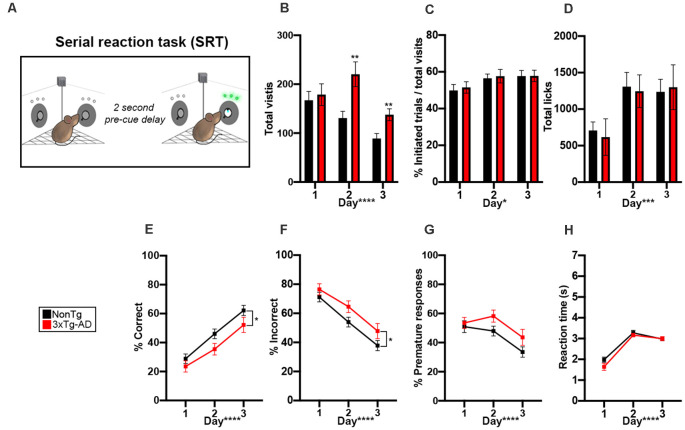
3xTg-AD mice showed impairments in attention in the Serial reaction time (SRT) attention tasks. **(A)** During the 6 days of the SRT task, animals were required to enter an assigned corner and nosepoke to initiate a trial. Then, a 2-s pre-cue delay was initiated and animals needed to learn to wait for the cue illumination. A green LED illuminated in one of the two noseports and the animal had 7 s to extinguish the LED with a nosepoke. A correct response resulted in access to water, while a premature (during 2-s delay) or incorrect response reset the trial and the animal was required to leave the corner before initiating a new trial; corner not drawn to scale. **(B)** 3xTg-AD mice made more total visits to all corners on day 2 (*p* < 0.01) and 3 (*p* < 0.01) of testing. Total visits went down across the 3 days of testing (*p* < 0.0001). **(C,D)** The number of initiated trials (*p* < 0.05) and total licks (*p* < 0.001) went up across the 3 days of testing. **(E)** % correct increased throughout the testing days (*p* < 0.0001), indicating learning. We found that 3xTg-AD mice had a significantly lower % correct than NonTg counterparts (*p* < 0.05). **(F)** % incorrect decreased throughout the testing days (*p* < 0.0001), indicating learning. We found that 3xTg-AD mice had a significantly higher % incorrect than NonTg counterparts (*p* < 0.05). **(G,H)** % premature responses decreased (*p* < 0.0001) and reaction time to extinguish the LED (*p* < 0.0001) increased across the 3 days. Data are presented as means ± SEM. *p < 0.05, **p < 0.01, ***p < 0.001, ****p < 0.0001.

To determine whether animals showed impairments in attention, increased impulsivity, or delayed reaction time, we next tested mice in the SRT attention task (Figure 4A). We first analyzed total visits and found a significant genotype by day interaction (*F*_(2,56)_ = 5.182, *p* < 0.01; Figure 4B). *Post hoc* analysis revealed that 3xTg-AD made more corner visits than NonTg mice on days 2 (*p* < 0.01) and 3 (*p* < 0.01). We found a main effect of the day for number of initiated trials in the correct corner during SRT, illustrating increased trial initiation per day (*F*_(2,56)_ = 4.711, *p* < 0.05; Figure 4C). We also found a main effect of day for total licks (*F*_(2,56)_ = 8.671, *p* < 0.001; Figure 4D), where the number of licks increased by the day. For % correct, we found a significant main effect of the day (*F*_(1,56)_ = 44.639, *p* < 0.0001, Figure 4E), illustrating learning across SRT testing days. We also found a main effect of genotype (F_1, 56)_ = 4.751, *p* < 0.05; Figure 4E), where the 3xTg-AD had a significantly lower % correct than the NonTg mice. Consistently, we found a main effect of day for % incorrect visits (*F*_(1,56)_ = 44.639, *p* < 0.0001, Figure 4F), where % incorrect went down across the SRT days, further illustrating learning. We also found a main effect of genotype (*F*_(1,56)_ = 4.751, *p* < 0.05) where the 3xTg-AD had a significantly higher % incorrect than NonTg mice. Failure to wait during the 2 s pre-cue delay between trial initiation and cue onset indicates impulsive behavior; when we measured % premature responses, we found a significant main effect of day (*F*_(1,56)_ = 11.169, *p* < 0.0001, Figure 4G), indicating that premature responses during the 2 s pre-cue delay went down across the SRT days. Notably, we found no significant genotype differences for premature responses (Figure 4G). Lastly, we measured reaction time to extinguish the LED and found no significant differences between the two genotypes (Figure 4H). Collectively, these results show attention deficits in the 3xTg-AD mice.

### 3xTg-AD Mice Showed Impairments in Working Memory During the Place Avoidance Task

**Figure 5:**
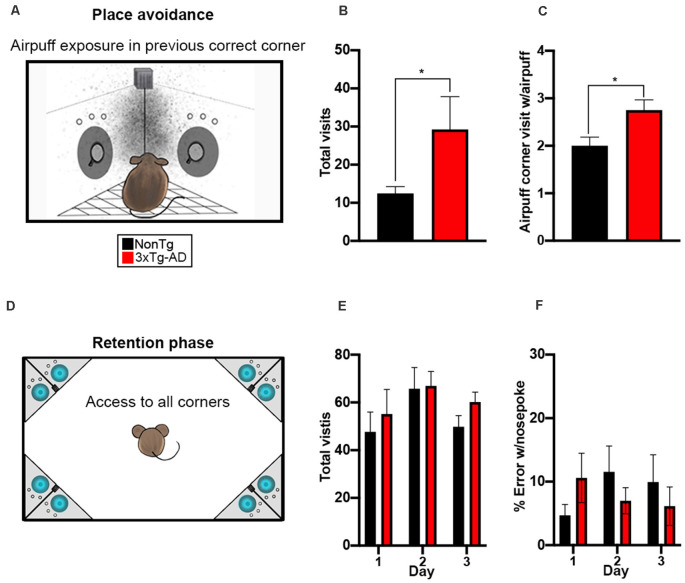
3xTg-AD mice show impairments in working memory during the place avoidance task. **(A)** For a 24-h period, entry into the assigned corner with a nosepoke resulted in an airpuff (~0.8 bar, 1 s airpuff); cage not drawn to scale. **(B,C)** We found that 3xTg-AD mice made more total visits (*p* < 0.05) and more visits to the airpuff corner with a nosepoke (*p* < 0.05) than the NonTg mice. **(D)** Mice were removed from the IntelliCage after the airpuff exposure and placed in a standard cage for 24 h, then returned to the IntelliCage to assess memory and extinction by measuring corner visits to the previously assigned airpuff corner; cage not drawn to scale. **(E,F)** No significant differences in total visits or % error with a nosepoke (i.e., visiting the airpuff corner from exposure) were detected. Data are presented as means ± SEM. *p < 0.05.

In the final phase of the IntelliCage, we tested all mice in a place avoidance learning task to measure both working and contextual memory (Figure 5A). During the 24-h period of airpuff exposure, entry into the correct corner from the SRT phase and a nosepoke resulted in an airpuff. We found a significant difference for total visits between the two genotypes (*t*_(28)_ = 2.302, *p* < 0.05, Figure 5B), where 3xTg-AD mice made more total visits than the NonTg mice. We also found a significant genotype difference for nosepokes within the airpuff corners (*t*_(28)_ = 2.642, *p* < 0.05, Figure 5C), where the 3xTg-AD mice entered the airpuff corner and nosepoked significantly more than the NonTg mice, illustrating deficits in working memory. After the 24-h airpuff exposure, mice were removed from the IntelliCage and placed in a standard cage for 24 h. The mice were then returned to the IntelliCage to assess memory and extinction by measuring corner visits with nosepokes to the previously assigned airpuff corner; all corners had water accessible in this phase (Figure 5D). We found no significant differences in total visits or in % error with nosepoke for the 3-day retention phase between the 3xTg-AD and NonTg mice (Figures 5E,F). In conclusion, the 3xTg-AD mice enter the airpuff corner and nosepoke significantly more illustrating working memory errors but remember the airpuff corner equally to NonTg mice after a 24-h delay.

### 3xTg-AD Mice Unable to Complete the IntelliCage Adaptation Phases Showed Increased Body Weight and a Reduced Brain to Body Ratio

Upon completion of IntelliCage testing, all mice were weighed and subsequently euthanized, with brains weighed and collected for neuropathological assessment. We first analyzed body, brain, and brain/body weight ratios to determine differences between NonTg (*n* = 23) and 3xTg-AD (*n* = 21). This included all mice, regardless of whether they completed or did not complete the IntelliCage testing (Table 2). We found a significant difference in body weight (*t*_(42)_ = 2.701, *p* < 0.01), where the 3xTg-AD mice weighed significantly more than the NonTg mice. Next, we measured brain weight and found a highly significant difference (*t*_(40)_ = 10.08, *p* < 0.0001), where 3xTg-AD mice have a lower brain weight than NonTg counterparts. We then examined the brain/body ratio and found a highly significant difference (*t*_(40)_ = 5.293, *p* < 0.0001), where the 3xTg-AD mice have a lower brain/body ratio than NonTg mice. Next, we examined whether these morphometric disparities persisted when comparing the NonTg (*n* = 18) and 3xTg-AD (*n* = 12) mice who were able to complete the IntelliCage tasks. There were no genotype differences for bodyweight of mice that completed the IntelliCage tasks. We did however find a significant difference for brain weight (*t*_(26)_ = 7.140, *p* < 0.0001), where 3xTg-AD mice had a lower brain weight than the NonTg mice. We also found a significant difference in brain/body ratio (*t*_(26)_ = 3.535, *p* < 0.01), where the 3xTg-AD mice had a significantly lower ratio than NonTg mice. Lastly, we examined body and brain weight differences in 3xTg-AD mice that completed the IntelliCage tasks (complete; *n* = 12) compared to those that could not complete adaptation phases (incomplete; *n* = 9). We found a significant difference in body weight (*t*_(19)_ = 2.970, *p* < 0.01), where 3xTg-AD complete mice weighed less than the 3xTg-AD incomplete mice. There were no significant differences for brain weight between the 3xTg-AD complete and incomplete mice. However, we found a significant difference in brain/body ratio (*t*_(19)_ = 3.396, *p* < 0.01), where 3xTg-AD complete mice had a higher brain/body ratio than the 3xTg-AD incomplete mice. These results show a difference in body and brain weight between NonTg and 3xTg-AD mice and suggest that brain weight and brain/body ratio may be a factor resulting in mice being unable to complete the IntelliCage tasks.

**Table 2:** Body and brain weight differences between 3xTg-AD and NonTg mice.

	All subjects		
Genotype	Body weight (g)	Brain weight (g)	Brain/Body weight ratio
NonTg (*n* = 23)	27.14 ± 0.64	0.485 ± 0.003	0.018 ± 0.0004
3xTg-AD (*n* = 21)	30.12 ± 0.92	0.441 ± 0.004	0.015 ± 0.0004
*p*-value	0.0099**	<0.0001****	<0.0001****
Genotype		**IntelliCage Complete**	
NonTg (*n* = 18)	26.71 ± 0.76	0.485 ± 0.003	0.018 ± 0.0005
3xTg-AD (*n* = 12)	28.12 ± 0.91	0.442 ± 0.006	0.016 ± 0.0004
*p*- value	0.250	<0.0001****	0.0016**
Testing		**IntelliCage 3xTg-AD**	
Complete (*n* = 12)	28.12 ± 0.91	0.442 ± 0.006	0.016 ± 0.0004
Incomplete (*n* = 9)	32.79 ± 1.35	0.440 ± 0.004	0.014 ± 0.0006
*p*-value	0.008**	0.828	0.003**

When including all subjects, 3xTg-AD mice show a significantly higher body weight and reduced brain weight compared to NonTg mice. The brain/body ratio is significantly lower for the 3xTg-AD mice. Two NonTg mice were excluded during place preference, bringing the *n* = 18. When including those that completed IntelliCage testing, 3xTg-AD mice show a significantly reduced brain and brain/body weight ratio than NonTg mice. When comparing 3xTg-AD mice that completed the IntelliCage testing versus those that did not (incomplete), we found a significantly higher body weight and a lower brain/body ratio for the incomplete. Data reported as Mean ± SEM. **p < 0.01, ****p < 0.0001.

### 3xTg-AD Mice That Were Unable to Complete the IntelliCage Adaptation Phases Showed Increased Insoluble Aß_40_ Levels and Decreased Insoluble Aß_42_ Levels

**Figure 6:**
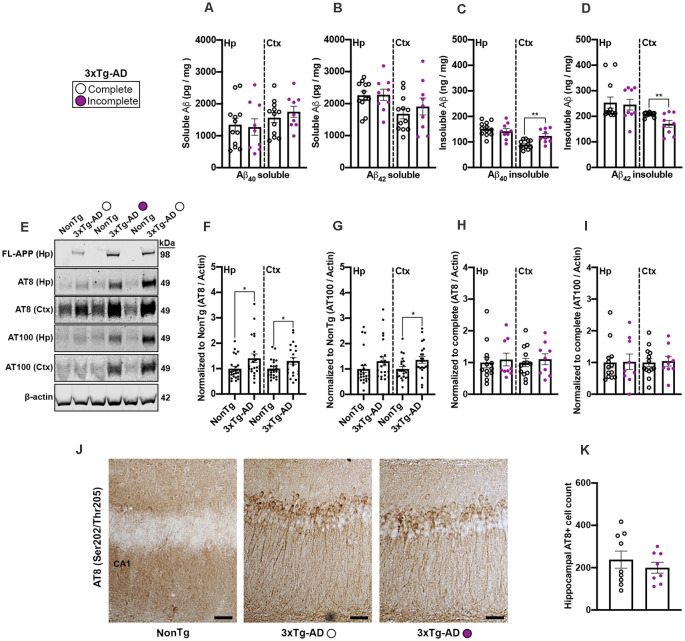
3xTg-AD mice that were unable to complete the IntelliCage adaptation phases showed increased insoluble Aß_40_ levels and decreased insoluble Aß_42_ levels. **(A,B)** No significant differences of soluble Aß_40–__42_ in the hippocampus (Hp) or cortex (Ctx) were found between 3xTg-AD that completed the IntelliCage tasks compared to 3xTg-AD incomplete mice. **(C)** We found that 3xTg-AD complete mice had a significantly lower level of insoluble Aß_40_ in the cortex than the 3xTg-AD incomplete mice (*p* < 0.01). **(D)** We found that 3xTg-AD complete mice had a significantly higher level of insoluble Aß_42_ in the cortex than the 3xTg-AD incomplete mice (*p* < 0.01). **(E)** Representative western blots of human APP, phospho(p) Ser202/Thr205 (AT8), Thr212/Ser214 (AT100), and ß-actin loading control. **(F)** 3xTg-AD mice showed higher expression of AT8 in the Hp and Ctx than NonTg mice. **(G)** 3xTg-AD mice show higher expression of AT100 in the Ctx than NonTg mice. **(H,I)** No significant difference was detected in AT8 or AT100 in the Hp and Ctx of 3xTg-AD complete compared to the 3xTg-AD incomplete mice**. (J)** Photomicrographs depicting the Hp Cornu Ammonis 1 (CA1) region stained for AT8+ cells. Scale bar = 50 μm. NonTg mice did not show detectable AT8+ cells in CA1 of the Hp. **(K)** No significant differences in AT8+ cell count were detected between 3xTg-AD complete compared to the 3xTg-AD incomplete mice. Data are presented as means ± SEM. *p < 0.05. **p < 0.01.

To determine whether common AD-like neuropathology markers could help explain why 3xTg-AD mice completed (complete; *n* = 12) or did not complete (incomplete; *n* = 9) IntelliCage tasks, we measured soluble and insoluble fractions of Aβ_40–42_ from hippocampal and cortical samples *via* ELISA. We found no significant differences for both soluble Aβ_40_ and Aβ_42_ levels for the 3xTg-AD complete compared to the 3xTg-AD incomplete mice (Figures 6A,B). We did however find a significant difference in the levels of cortical insoluble Aβ_40_ (*t*_(19)_ = 3.556, *p* < 0.01, Figure 6C), where the 3xTg-AD complete mice had a lower level than their incomplete counterparts. We also found a significant difference in the levels of cortical insoluble Aβ_42_ (*t*_(19)_ = 3.124, *p* < 0.01, Figure 6D), where the 3xTg-AD complete mice had a higher level than the incomplete mice. Next, to determine whether tau phosphorylation may contribute to 3xTg-AD mice not being able to complete the IntelliCage tasks, we performed western blots (Figure 6E) for serine (Ser) and threonine (Thr) tau phosphorylation sites Ser202/Thr205 (AT8) and Thr212/Ser214 (AT100); AT8 and AT100 are detectable *via* western blot by 6 months of age in 3xTg-AD mice (Oh et al., 2010; Parachikova et al., 2010). We also verified the presence of human APP *via* immunoblot using these same protein homogenates (Figure 6E). Quantitative analysis revealed a significant elevation of AT8 in both the hippocampus (*t*_(44)_ = 2.159, *p* < 0.05; 6E, F) and cortex (*t*_(43)_ = 2.057, *p* < 0.05; Figures 6E,F) of 3xTg-AD mice compared to NonTg mice. For AT100, we found a significant elevation in the cortex of 3xTg-AD mice compared to NonTg mice (*t*_(42)_ = 2.246, *p* < 0.05; Figures 6E,G). Next, we analyzed AT8 and AT100 immunoblot expression between the 3xTg-AD complete vs incomplete samples and found no significant differences in either the hippocampus or cortex (Figures 6E,H,I). Lastly, we performed immunostaining for AT8 and found consistent results showing that AT8+ cells in the hippocampus were not statistically different between the 3xTg-AD complete versus incomplete mice (Figures 6J,K). Collectively, these results suggest that brain weight and fractions of cortical insoluble Aβ_40–42_ may be associated with mice not being able to complete the adaptation phases in the IntelliCage.

## Discussion

Our results highlight, for the first time, that female 3xTg-AD mice show cognitive deficits in various tasks of the automated IntelliCage system. During the adaptation phases, only 57.14% of the 3xTg-AD mice were able to learn to drink water accessed through the noseport, while 86.96% of the NonTg mice drank and made it past all adaptation phases. Our data showed that the 3xTg-AD mice made fewer corner visits compared to NonTg mice during the first day of introduction into the IntelliCage. As testing progressed, 3xTg-AD mice consistently entered corners more frequently with the purpose to drink than the NonTg mice, which resulted in a higher percentage of correct corner visits during the reversal place preference phase. This suggests that during the place preference phases, the 3xTg-AD mice’s sole purpose to enter a corner was to drink while NonTg mice entered corners to both obtain water and explore, thereby explaining this discrepancy in increased percent correct visits of 3xTg-AD mice in these phases of the IntelliCage. Previous reports show that increased body weight is associated with increased water consumption in transgenic mice (Bachmanov et al., 2002). While the body weight assessment at the end of our study showed the 3xTg-AD mice weighed significantly more than the NonTg mice, consistent with previous studies (Robison et al., 2020), 3xTg-AD mice that completed the adaptation phases were not significantly heavier than their NonTg counterparts. Additionally, water consumption between NonTg and 3xTg-AD mice was not significantly different during any of the IntelliCage tasks. Alternatively, the observed reduced exploratory behavior in 3xTg-AD mice could instead be an indicator of increased anxiety in 3xTg-AD mice (Giménez-Llort et al., 2013). Indeed, neophobia and anxiety can be measured immediately after introducing animals into a new environment, such as the IntelliCage (Kiryk et al., 2020). It is notable that 3xTg-AD mice only showed reduced exploratory behavior on day 1, as they likely acclimated to their new environment on subsequent days given equal corner visits compared to NonTg mice. These findings are consistent with previous reports showing that 3xTg-AD mice exhibit reduced exploration due to increased anxiety (Sterniczuk et al., 2010).

Executive functions such as attention and impulsivity, as well as working memory, are mediated by areas of the prefrontal cortex (Voikar et al., 2018). In AD, while the main focus has been on learning and memory deficits early in the progression of the disease, attentional dysfunction is also present as the disease evolves (Perry and Hodges, 1999; Berardi et al., 2005). The SRT and place avoidance tasks in the IntelliCage were used to assess these domains. The 3xTg-AD mice showed a lower percentage correct and a higher percent incorrect in the SRT task than the NonTg mice, which is consistent with previous reports showing attention deficits in this mouse model (Romberg et al., 2011). Notably, the number of total visits to all corners was significantly increased in 3xTg-AD particularly on day 2 and 3 of SRT testing; however, the number of trials initiated in the assigned corner were similar between the NonTg and 3xTg-AD mice on days 1–3. Given that the SRT task relies on mice to initiate a trial in only the assigned corner to gain water access, this suggests that the 3xTg-AD were likely entering other corners in search of water given that their percent correct was lower than the NonTg mice. Similarly, 3xTg-AD mice exhibited a higher number of working memory errors in the place avoidance task compared to their NonTg counterparts, also consistent with previous reports and illustrating deficits mediated by the prefrontal cortex (Stevens and Brown, 2015; Li et al., 2020).

Our data shows that 3xTg-AD mice had lower brain weights than their NonTg counterparts. This is consistent with a recent report showing that both male and female 3xTg-AD mice have lower brain mass than NonTg mice (Li et al., 2020). The findings that more 3xTg-AD mice were not able to successfully advance past the adaptation phases and had reduced brain weights are consistent with a report illustrating that brain mass may be an indicator of cognitive capacities (Perepelkina et al., 2020). Future work will focus on whether such brain weight differences in 3xTg-AD are the result of atrophy, or if there may be developmental issues in this transgenic mouse model contributing to such discrepancies. Further neuropathological analysis of AD-like pathology markers revealed that 3xTg-AD mice that completed the IntelliCage tasks had a lower level of cortical insoluble Aβ_40_ and a higher level of insoluble Aβ_42_. A previous report found that female 3xTg-AD mice’s insoluble Aβ_40_ and Aβ_42_ levels increased significantly with age from 6–12 months (Oddo et al., 2003). However, insoluble Aβ_40_ levels have been shown to increase early and play a mechanistic role in the progression of AD (Wang et al., 1999). Given that 3xTg-AD mice in our study were between 7–9 months of age when IntelliCage testing commenced suggests that insoluble Aβ_40_ levels may be an early predictor of performance in these mice and that Aβ_42_ levels may become more relevant at later ages. When examining phosphorylation markers of tau, we found significant elevations of phosphorylation at Ser202/Thr205 (AT8) in the hippocampus and cortex of 3xTg-AD mice compared to NonTg that completed the IntelliCage tasks. For phosphorylated tau at Thr212/Ser214 (AT100), we only found significant differences between NonTg and 3xTg-AD in the cortex. This is not surprising, as tau phosphorylation at AT8 is widespread in the hippocampus by 6 months of age, while tau phosphorylated at AT100 is more widespread in the hippocampus by 12 months (Oh et al., 2010; Parachikova et al., 2010). These results suggest that phosphorylated tau may play a role in differences observed between NonTg and 3xTg-AD mice. However, we did not find any significant phosphorylated tau differences in 3xTg-AD mice that completed the IntelliCage tasks compared to those that failed to pass the adaptation phases. Given the age of the 3xTg-AD mice in the current study and the limited amount of tau pathology at this age, it is not likely that tau was a driving factor for the 3xTg-AD that completed versus those that did not complete the tasks.

In summary, this work is the first to report behavioral deficits in 3xTg-AD mice on various tasks of the IntelliCage. We found that 3xTg-AD mice explore less on the first day in the novel environment, indicating increased anxiety, in addition to deficits in attention and working memory in later tasks. More than one-third of the 3xTg-AD mice did not complete the IntelliCage tasks and showed differences in brain weight, brain-to-body weight ratios, and insoluble Aβ_40–42_. Collectively, these results fill in the gap of a much-needed area of research, testing a widely used mouse model of AD in the IntelliCage, and will inform scientists of important factors to consider when testing the 3xTg-AD mouse in this automated behavioral phenotyping system.

## Data Availability Statement

The raw data supporting the conclusions of this article will be made available by the authors, without undue reservation.

## Ethics Statement

The animal study was reviewed and approved by IACUC.

## Author Contributions

WW: experimental design, IntelliCage testing, ELISA, figure creation, writing and editing the manuscript. IM: IntelliCage testing, algorithm development and data extraction, writing and editing the manuscript. ST: figure creation, animal harvesting, immunohistochemistry, writing and editing the manuscript. AD: IntelliCage testing assistance, animal harvesting assistance, westernblotting, immunohistochemistry, and editing the manuscript. AV: animal harvesting assistance, image quantification, statistical analysis, figure creation, and editing the manuscript. RV: experimental design, funding, IntelliCage testing, statistical analysis, figure creation, writing and editing the manuscript. All authors contributed to the article and approved the submitted version.

## Conflict of Interest

The authors declare that the research was conducted in the absence of any commercial or financial relationships that could be construed as a potential conflict of interest.

## Publisher’s Note

All claims expressed in this article are solely those of the authors and do not necessarily represent those of their affiliated organizations, or those of the publisher, the editors and the reviewers. Any product that may be evaluated in this article, or claim that may be made by its manufacturer, is not guaranteed or endorsed by the publisher.

## References

[B1] Alzheimer’s association report (2021). 2021 Alzheimer’s disease facts and figures. Alzheimers Dement. 17, 327–406. 10.1002/alz.12328 33756057

[B2] Ajonijebu D. C. Abboussi O. Mabandla M. V. Daniels W. M. U. (2018). Differential epigenetic changes in the hippocampus and prefrontal cortex of female mice that had free access to cocaine. Metab. Brain Dis. 33, 411–420. 10.1007/s11011-017-0116-z 28963688

[B3] Allen B. Ingram E. Takao M. Smith M. J. Jakes R. Virdee K.. (2002). Abundant tau filaments and nonapoptotic neurodegeneration in transgenic mice expressing human P301S tau protein. J. Neurosci. 22, 9340–9351. 10.1523/JNEUROSCI.22-21-09340.2002 12417659 PMC6758022

[B4] Alz Forum (2021). Available online at: https://www.alzforum.org/research-models/alzheimers-disease. Accessed February 25, 2021.

[B5] Augustinack J. C. Schneider A. Mandelkow E.-M. Hyman B. T. (2002). Specific tau phosphorylation sites correlate with severity of neuronal cytopathology in Alzheimer’s disease. Acta Neuropathol. 103, 26–35. 10.1007/s004010100423 11837744

[B6] Bachmanov A. A. Reed D. R. Beauchamp G. K. Tordoff M. G. (2002). Food intake, water intake and drinking spout side preference of 28 mouse strains. Behav. Genet. 32, 435–443. 10.1023/a:1020884312053 12467341 PMC1397713

[B7] Bakota L. Brandt R. (2016). Tau biology and tau-directed therapies for Alzheimer’s disease. Drugs 76, 301–313. 10.1007/s40265-015-0529-0 26729186 PMC4757605

[B8] Bateman R. (2015). Alzheimer’s disease and other dementias: advances in 2014. Lancet Neurol. 14, 4–6. 10.1016/S1474-4422(14)70301-1 25496880

[B10] Berardi A. M. Parasuraman R. Haxby J. V. (2005). Sustained attention in mild Alzheimer’s disease. Dev. Neuropsychol. 28, 507–537. 10.1207/s15326942dn2801_4 15992254 PMC2383280

[B11] Borchelt D. R. Ratovitski T. van Lare J. Lee M. K. Gonzales V. Jenkins N. A.. (1997). Accelerated amyloid deposition in the brains of transgenic mice coexpressing mutant presenilin 1 and amyloid precursor proteins. Neuron 19, 939–945. 10.1016/s0896-6273(00)80974-5 9354339

[B12] Braak H. Alafuzoff I. Arzberger T. Kretzschmar H. Del Tredici K. (2006). Staging of Alzheimer disease-associated neurofibrillary pathology using paraffin sections and immunocytochemistry. Acta Neuropathol. 112, 389–404. 10.1007/s00401-006-0127-z 16906426 PMC3906709

[B13] Braak H. Braak E. (1991). Neuropathological stageing of Alzheimer-related changes. Acta Neuropathol. 82, 239–259. 10.1007/BF00308809 1759558

[B14] Dave N. Vural A. S. Piras I. S. Winslow W. Surendra L. Winstone J. K.. (2021). Identification of retinoblastoma binding protein 7 (Rbbp7) as a mediator against tau acetylation and subsequent neuronal loss in Alzheimer’s disease and related tauopathies. Acta Neuropathol. 142, 279–294. 10.1007/s00401-021-02323-1 33978814 PMC8270842

[B15] Dell’Omo G. Ricceri L. Wolfer D. P. Poletaeva I. I. Lipp H. (2000). Temporal and spatial adaptation to food restriction in mice under naturalistic conditions. Behav. Brain Res. 115, 1–8. 10.1016/s0166-4328(00)00234-5 10996402

[B16] Di Benedetto G. Burgaletto C. Carta A. R. Saccone S. Lempereur L. Mulas G.. (2019). Beneficial effects of curtailing immune susceptibility in an Alzheimer’s disease model. J. Neuroinflammation 16:166. 10.1186/s12974-019-1554-9 31409354 PMC6693231

[B17] Giménez-Llort L. Rivera-Hernández G. Marin-Argany M. Sánchez-Quesada J. L. Villegas S. (2013). Early intervention in the 3xTg-AD mice with an amyloid ?-antibody fragment ameliorates first hallmarks of Alzheimer disease. mAbs 5, 665–677. 10.4161/mabs.25424 23884018 PMC3851220

[B18] Hall A. M. Roberson E. D. (2012). Mouse models of Alzheimer’s disease. Brain Res. Bull. 88, 3–12. 10.1016/j.brainresbull.2011.11.017 22142973 PMC3546481

[B19] Honjo K. Black S. E. Verhoeff N. P. L. G. (2012). Alzheimer’s disease, cerebrovascular disease and the β-amyloid cascade. Can J. Neurol. Sci. 39, 712–728. 10.1017/s0317167100015547 23227576

[B20] Jankowsky J. L. Fadale D. J. Anderson J. Xu G. M. Gonzales V. Jenkins N. A.. (2004). Mutant presenilins specifically elevate the levels of the 42 residue beta-amyloid peptide *in vivo*: evidence for augmentation of a 42-specific gamma secretase. Hum. Mol. Genet. 13, 159–170. 10.1093/hmg/ddh019 14645205

[B21] Kiryk A. Janusz A. Zglinicki B. Turkes E. Knapska E. Konopka W.. (2020). IntelliCage as a tool for measuring mouse behavior - 20 years perspective. Behav. Brain Res. 388:112620. 10.1016/j.bbr.2020.112620 32302617

[B22] LaFerla F. M. Green K. N. (2012). Animal models of Alzheimer disease. Cold Spring Harb. Perspect. Med. 2:a006320. 10.1101/cshperspect.a006320 23002015 PMC3543097

[B23] Lane C. A. Hardy J. Schott J. M. (2018). Alzheimer’s disease. Eur. J. Neurol. 25, 59–70. 10.1111/ene.13439 28872215

[B24] Lee K. Kobayashi Y. Seo H. Kwak J.-H. Masuda A. Lim C.-S.. (2015). Involvement of cAMP-guanine nucleotide exchange factor II in hippocampal long-term depression and behavioral flexibility. Mol. Brain 8:38. 10.1186/s13041-015-0130-1 26104314 PMC4477293

[B25] Li T. Jiao J.-J. Su Q. Hölscher C. Zhang J. Yan X.-D.. (2020). A GLP-1/GIP/Gcg receptor triagonist improves memory behavior, as well as synaptic transmission, neuronal excitability and Ca2+ homeostasis in 3xTg-AD mice. Neuropharmacology 170:108042. 10.1016/j.neuropharm.2020.108042 32147454

[B26] Lipp H.-P. (2005). High-throughput and automated behavioural screening of normal and genetically modified mice. Buisness Brief: Future Drug Dis. 5, 1–5.

[B27] Lipp H.-P. Litvin O. Galsworthy M. Vyssotski D. Vyssotski A. Zinn P.. (2005). Automated behavioral analysis of mice using INTELLICAGE: inter-laboratory comparisons and validation with exploratory behavior and spatial learning. Proc. Measuring Behav. 5, 66–69.

[B28] Luna-Muñoz J. Chávez-Macías L. García-Sierra F. Mena R. (2007). Earliest stages of tau conformational changes are related to the appearance of a sequence of specific phospho-dependent tau epitopes in Alzheimer’s disease. J. Alzheimers Dis. 12, 365–375. 10.3233/jad-2007-12410 18198423

[B29] Masuda A. Kobayashi Y. Itohara S. (2018). Automated, long-term behavioral assay for cognitive functions in multiple genetic models of Alzheimer’s disease, using intellicage. J. Vis. Exp. 58009, 1–11. 10.3791/58009 30124661 PMC6126617

[B30] Masuda A. Kobayashi Y. Kogo N. Saito T. Saido T. C. Itohara S. (2016). Cognitive deficits in single App knock-in mouse models. Neurobiol. Learn. Mem. 135, 73–82. 10.1016/j.nlm.2016.07.001 27377630

[B31] Mifflin M. A. Winslow W. Surendra L. Tallino S. Vuraal A. Velazquez R. (2021). Sex differences in the IntelliCage and the Morris water maze in the APP/PS1 mouse model of amyloidosis. Neurobiol. Aging 101, 130–140. 10.1016/j.neurobiolaging.2021.01.018 33610962 PMC8122060

[B32] O’Brien R. J. Wong P. C. (2011). Amyloid precursor protein processing and Alzheimer’s disease. Annu. Rev. Neurosci. 34, 185–204. 10.1146/annurev-neuro-061010-113613 21456963 PMC3174086

[B33] Oddo S. Caccamo A. Shepherd J. D. Murphy M. P. Golde T. E. Kayed R.. (2003). Triple-transgenic model of Alzheimer’s disease with plaques and tangles: intracellular Aβ and synaptic dysfunction. Neuron 39, 409–421. 10.1016/s0896-6273(03)00434-3 12895417

[B34] Oh K.-J. Perez S. E. Lagalwar S. Vana L. Binder L. Mufson E. J. (2010). Staging of Alzheimer’s pathology in triple transgenic mice: a light and electron microscopic analysis. Int. J. Alzheimers Dis. 2010:780102. 10.4061/2010/780102 20798886 PMC2925282

[B460] Parachikova A. Visilevko V. Cribbs D. H. LaFerla F. M. Green K. N. (2010). Reductions in amyloid-β-derived neuroinflammation, with minocycline, restore cognition but do not significantly affect tau hyperphosphorylation. J. Alzheimer’s Dis. 21, 527–542. 10.3233/JAD-2010-100204 20555131 PMC4085054

[B35] Perepelkina O. V. Tarasova A. Y. Ogienko N. A. Lil’p I. G. Poletaeva I. I. (2020). Brain weight and cognitive abilities of laboratory mice. Biol. Bull Rev. 10, 91–101. 10.1134/S2079086420020061

[B36] Perry R. J. Hodges J. R. (1999). Attention and executive deficits in Alzheimer’s disease: a critical review. Brain 122, 383–404. 10.1093/brain/122.3.383 10094249

[B37] Reiserer R. S. Harrison F. E. Syverud D. C. McDonald M. P. (2007). Impaired spatial learning in the APPSwe + PSEN1DeltaE9 bigenic mouse model of Alzheimer’s disease. Genes Brain Behav. 6, 54–65. 10.1111/j.1601-183X.2006.00221.x 17233641

[B38] Robison L. S. Gannon O. J. Thomas M. A. Salinero A. E. Abi-Ghanem C. Poitelon Y.. (2020). Role of sex and high-fat diet in metabolic and hypothalamic disturbances in the 3xTg-AD mouse model of Alzheimer’s disease. J. Neuroinflammation 17:285. 10.1186/s12974-020-01956-5 32993686 PMC7526387

[B420] Roda A. R. Esquerda-Canals G. Marti-Clua J. Villegas S. (2020). Congitive impairment in the 3xTg-AD mouse of Alzheimer’s disease is affected by Aβ-immunotherapy and cognitive stimulation. Pharmaceuticals 12:944. 10.3390/pharmaceutics12100944 33023109 PMC7601886

[B39] Romberg C. Mattson M. P. Mughal M. R. Bussey T. J. Saksida L. M. (2011). Impaired attention in the 3xTgAD mouse model of Alzheimer’s disease: rescue by donepezil (Aricept). J. Neurosci. 31, 3500–3507. 10.1523/JNEUROSCI.5242-10.2011 21368062 PMC3066152

[B40] Ryan D. Koss D. Porcu E. Woodcock H. Robinson L. Platt B.. (2013). Spatial learning impairments in PLB1Triple knock-in Alzheimer mice are task-specific and age-dependent. Cell. Mol. Life Sci. 70, 2603–2619. 10.1007/s00018-013-1314-4 23535719 PMC11113905

[B41] Sadigh-Eteghad S. Sabermarouf B. Majdi A. Talebi M. Farhoudi M. Mahmoudi J. (2015). Amyloid-beta: a crucial factor in Alzheimer’s disease. Med. Princ. Pract. 24, 1–10. 10.1159/000369101 25471398 PMC5588216

[B42] Sterniczuk R. Antle M. C. Laferla F. M. Dyck R. H. (2010). Characterization of the 3xTg-AD mouse model of Alzheimer’s disease: part 2. behavioral and cognitive changes. Brain Res. 1348, 149–155. 10.1016/j.brainres.2010.06.011 20558146

[B43] Stevens L. M. Brown R. E. (2015). Reference and working memory deficits in the 3xTg-AD mouse between 2 and 15-months of age: a cross-sectional study. Behav. Brain Res. 278, 496–505. 10.1016/j.bbr.2014.10.033 25446812

[B44] Velazquez R. Ferreira E. Knowles S. Fux C. Rodin A. Winslow W.. (2019a). Lifelong choline supplementation ameliorates Alzheimer’s disease pathology and associated cognitive deficits by attenuating microglia activation. Aging Cell 18:e13037. 10.1111/acel.13037 31560162 PMC6826123

[B45] Velazquez R. Ferreira E. Winslow W. Dave N. Piras I. S. Naymik M.. (2019b). Maternal choline supplementation ameliorates Alzheimer’s disease pathology by reducing brain homocysteine levels across multiple generations. Mol. Psychiatry 25, 2620–2629. 10.1038/s41380-018-0322-z 30622336 PMC6697226

[B46] Velazquez R. Meechoovet B. Ow A. Foley C. Shaw A. Smith B.. (2019c). Chronic dyrk1 inhibition delays the onset of AD-like pathology in 3xTg-AD mice. Mol. Neurobiol. 56, 8364–8375. 10.1007/s12035-019-01684-9 31240602

[B47] Velazquez R. Shaw D. M. Caccamo A. Oddo S. (2016). Pim1 inhibition as a novel therapeutic strategy for Alzheimer’s disease. Mol. Neurodegener 11:52. 10.1186/s13024-016-0118-z 27412291 PMC4944476

[B48] Voikar V. Krackow S. Lipp H.-P. Rau A. Colacicco G. Wolfer D. P. (2018). Automated dissection of permanent effects of hippocampal or prefrontal lesions on performance at spatial, working memory and circadian timing tasks of C57BL/6 mice in IntelliCage. Behav. Brain Res. 352, 8–22. 10.1016/j.bbr.2017.08.048 28927717 PMC6102415

[B49] Wang J. Dickson D. W. Trojanowski J. Q. Lee V. M. (1999). The levels of soluble versus insoluble brain Abeta distinguish Alzheimer’s disease from normal and pathologic aging. Exp. Neurol. 158, 328–337. 10.1006/exnr.1999.7085 10415140

